# The comparison of perceived stress in idiopathic thrombocytopenic purpura patients referred to Seyed Al-Shohada Hospital with healthy people in Isfahan, Iran, 2013

**Published:** 2015-04-01

**Authors:** Zeinab Hemati, Davood Kiani

**Affiliations:** 1 PhD Candidate of Nursing, School of Nursing and Midwifery, Isfahan University of Medical Sciences, Isfahan, Iran; 2 BSc of Nursing, Shahrekord University of Medical Sciences, Shahrekord, Iran

**Keywords:** Perceived stress, Idiopathic thrombocytopenic purpura, Iran

## Abstract

**Background**: Mental stress and daily crises comprise a part of physical and mental threats. Perceived stress is a physical and mental threat, as well. Perceived stress is a psychological process during which the individual considers his/ her physical and psychological welfare as being threatened. Since idiopathic thrombocytopenic purpura (ITP) is one of the chronic diseases being able to affect patients' perceived stress, this study was conducted to compare perceived stress in ITP patients and healthy people.

**Materials and Methods:** This is a descriptive-comparative study with control and case groups. In this study, 64 ITP patients referring Seyed Al-Shohada Hospital and the same number of healthy individuals from the patients' neighborhood, as the control group, were selected randomly and compared. The Kohen Perceived Stress Standard Questionnaire was used to collect the data. The data were analyzed by SPSS and Student’s independent t-test, chi-square, and Mann-Whitney test.

**Results** **:** 64.1%, 59.4% and 53.1% of participants in case group were older than 35 years old, female and had elementary education. 78.1% of case group had severe perceived stress. 70.3% of participants in control group experienced mild perceived stress. Mann-Whitney test showed significant difference between two groups in level of stress (p<0.001).

Conclusion: In ITP patients, perceived stress was considerable. Planning interventional measures to determine stress-making agents and subside or at least control them is very essential.

## Introduction

 Platelets have an important role in stabilizing hemostasis. Idiopathic thrombocytopenic purpura (ITP) is one of the diseases in which platelets disturbance happens. The number and function of platelet are disturbed in this disease, leading to bleeding. Bleeding mostly happens in skin and mucosa. The most prevalent cause of thrombocytopenia is platelet destruction. ITP is a syndrome which is more frequently seen in spring and winter.^1^ It can be seen in acute and chronic forms. In its acute form, the number of platelets is higher than 150000 per mm after six months and is not found in recurrent form. In contrast, in the chronic form, the number of platelets remains at low level for more than six months.^2^

 The prevalence of ITP is 2 to 5 per 100000 in children and 5 to 6 per 100000 in adults. Moreover, 61% and 38% of ITP patients are women and men, respectively. In addition, 4-7 million people have chronic ITP worldwide. This disease is more prevalent in Australia and Canada.^3^^,^^4^ The most common complications of petechia comprise anemia and massive bleedings needing various treatments. Although the mortality rate of this disease is not high, patients and their families undergo a great deal of stress, fear of bleeding, economic problems and recurrent hospitalizations which affect their mood drastically.^5^^-^^7^

 Gastrointestinal bleeding and vast ecchymosis on skin and neck influence the patient's life greatly.^8^ Mental disorders such as depression, anxiety, reduction of interpersonal relations and daily activities due to tiredness lead to decrease their function. Shamefulness related to disease signs and restriction of personal communication is one of the main causes of patient's stress.^9^ Since this illness with severe complications can lead to anxiety and depression abruptly, diminishes patients' adjustment capabilities. Stress is an inevitable reality, and it is important how to manage it. The study of human's responses against stress-making situations shows that he reacts against risky symbols and threats. Coping reactions vary according to the stressful situation. Coping reactions vary in different stressful situations.

 Perceived stress is a psychological process during which the person perceives his/her own physical and psychological welfare as being threatened. In fact, stress impact depends on individuals' perception of conditions related to illness. A single situation which is supposed to be safe for someone can be accounted risky for the other. Therefore, those people who believe that they have supportive resources to ask them any necessary aid are less vulnerable against stress.^10^^-^^12^

 High expense of treatment, permanent family and patients' anxiety and long term hospitalizations lead to stress, helplessness, low self-esteem and self-confidence and social withdrawal and in ITP patients .^5^^,^^9^^,^^13^

 As a result, medical team should recognize vulnerable patients through qualified tests and exams. Then, they have to plan efficient interventions to minimize their psychological problems as much as possible. As a matter of fact, treatment efficacy can be improved by decreasing hospitalized patients. This study was done to compare perceived stress of ITP patients’ with healthy individuals. This will make a suitable footstep to utilize the results of this project practically by medical officials to improve patients' treatment process and reduce their perceived stress. It finally results in promoting patients' quality of life and health condition. 

## SUBJECTS AND METHODS

 This was a case-control study consisting of two groups of 64 each. Researcher received all necessary documents and preliminaries from professional officials.

 Then, they selected the patients having essential specifications to take part in the study. Inclusion criteria were being 20 to 70 years old, living in Isfahan city, being diagnosed at least for 6 months as a known case of the ITP through paramedical and medical tests (chronic disease is defined as an illness being diagnosed at least three months and disturbs patient's daily life and his/her quality of life). Other inclusion criteria comprised not to have any mental or cognitive problems and not to face any stress-making events in their life in recent months such as a relative death. Exclusion criteria were not willing to participate in the study.

 After describing study's goals to participants and receiving written consent, the researcher began completing the questionnaires. 64 control cases were selected randomly. Since the people living in patients' neighborhood have rather the same economic and social conditions, they were selected as the control to take part in the study. The process of this selection was as follows; the researcher referred to patients' neighbors randomly and explained study's goals to them. If their demographic data were close to patients and also they were willing to take part in the project, the questionnaire was completed for them.

 Perceived stress and ITP morbidity were the main variables and age, sex, educational level, duration of disease, morbidity and hospitalization frequencies were contextual variables. 

 Data collection method was Kohen Perceived Stress Questionnaire consisting of three transcripts.

They are used to measure general perceived stress during last months. This questionnaire measures thoughts, feelings about stress-inducing events and perceived stresses.

Kohen et al. calculated Cronbach’s alpha for this scale 84% and 86%. Scoring method of this questionnaire is based on Likert five-point scale. 0, 1, 2, 3, 4 scores were defined as never, seldom, sometimes, often and more than often, respectively. Four to 13 questions were scored diversely from never (4) up to more than often (0). The least score was 14 and the highest was 70. 

 In terms of the validity and constancy of data collection instrument, the questionnaire of perceived stress is a standard questionnaire whose scientific validity has been assessed in several researches.^14^^,^^15^ Collected data were analyzed by SPSS15. The comparison of age, sex and educational level between two groups was done by independent t-test, chi square and Mann-Whitney tests, respectively. Perceived stress comparison between two groups was analyzed by Mann-Whitney test, as well.

## Results

 The most studied cases, in both groups, were older than 35 years old (64.1% and 56.3% in case and control groups, respectively). Independent t-test did not show any significant difference in age between two groups (p>0.05).

59.4% and 70.3% in respectively control and case groups were women. There was no significant difference in gender, as well (p=0.19).

 Most of the cases studied up to elementary level (53.1% in case group). There was no significant difference between two groups in education level (p=0.43). Spearman coefficient test showed positive significant relationship between gender and perceived stress. Woman's perceived stress was higher than men's (r=0.19, p=0.02). 72.5% of the case group had severe level of stress, whereas 76.3% of the case group had average level of stress. Mann-Whitney test illustrated significant difference between two groups in perceived stress (p<0.001) (Table 1).

## Discussion

 Nowadays, positive-oriented psychology considers mental health specifically. This approach regards it as an optional condition of physical, social and mental health. Some of mental health indicators consist of absence of stress, anxiety and depression^16^ Stress is any external or internal change or stimulant which may cause disturbance in vital balance. It may lead to illness in some especial circumstances. In fact, anxiety is the feature of an unsuitable emotional condition derived from mental conflicts and pressures. It is typical characteristic is fear of future events.^17^ Some factors such as inability to solve problems, emotional-centred reactions, inability to control stress, negative attitudes, family conflicts and gender variables cause stress in individuals.^18^

 Perceived stress is one of the most constructs of Health Belief Model based on psychological learning theory. According to this model, the severity of perceived stress is one of the basic constructs anticipating the possibility of the strategy taken by individuals against stress-making situations. The severity of perceived stress is closely associated with individual's belief about the seriousness of stress. The individual may take qualified measures possibly after coming to this conclusion that stress has social, psychological and physical complications such as changing social relations, independence reduction, burden and pain, inability and even death.^19^

 Since most participants, in present study, were older than 36 and women, its results are rather similar to Susan et al study in which the age of most studied individuals was more than 45 years old and 77% were women.^13^ This confirmed that people caught by chronic diseases may be older than young ages. It confirms the results of present study.^20^^,^^21^ Since most of the patients had primary education level, they are more prone to disease complication and their negative impacts on their lifestyle. It makes them lose the chances to get a raise in various fields.^22^

 78% of ITP patients suffered from severe perceived stress because of recurrent bleeding and repeated long-term hospitalizations. The patients caught by chronic disease are exposed to low level of self-esteem and loss of control feeling. These will deteriorate their life quality and increase their depression, stress and physical problems.^23^

Makinson et al. found that fear; anger, stress and depression were seen after frequent bleedings.^9^ Vahedian Azimi et al. study (2012) showed that the average level of perceived stress in cardiac patients

was significantly higher than healthy people.^24^

**Table1:** The comparison of perceived stress in the two groups

**Group (Perceived stress)**	**Case**		**Control**	
**Low (14-32)**	number	Percentage	number	Percentage
	0	0	0	0
**Mild (33-51)**	14	21.9	45	70.3
**Severe (52-70)**	50	78.1	19	29.7
**Total **	64	100	64	100
**Mann-Whitney test result **	P<0.001			

As the studied cases were rather middle aged or old and had low level of education, focusing on stress and manipulating is more crucial. Education and individual ability to completely control the illness is very significant. Since chronic diseases are very important and have considerable impacts on patients mentally and socially, especially among ITP patients suffering from ITP complications and its sequence stress, treatment team should monitor patients' and their families’ stress closely and teach them preventive strategies.

 Low number of participants was the main limitation in this study. So, we suggest to conduct studies with larger sample size and to compare their findings with the present study's results.

## CONCLUSION

 It is suggested, according to present study's results, its sequence stress, treatment, that doctors, nurses and other medical professionals obtain wider attitude towards perceived stress and apply educational programs to decrease mental pressure in remedial plans provided for patients. This can lead to diminish their perceived stress, because qualified implementation of these plans prevents from mental, spiritual complications and decrease treatment costs which patients and their families may have to deal with. 
